# A swept-source optical coherence tomography study of the spectrum of laser pointer maculopathy

**DOI:** 10.1186/s40942-025-00683-4

**Published:** 2025-06-05

**Authors:** Magdy Moussa, Mahmoud Leila, Mohammed Fotouh Elashri, Ahmed Osama Hashem

**Affiliations:** 1 1Ophthalmology Department, Faculty of Medicine, Tanta University, El Bahr Street, Tanta Qism 2, Tanta, 31111 Gharbia Governorate Egypt; 2 Retina Department, Research Institute of Ophthalmology, Giza, Egypt; 3 Ophthalmology Department, Faculty of Medicine, Kafr El Sheikh University, Kafr El Sheikh, Egypt

**Keywords:** Hand-held laser pointer, Laser pointer maculopathy, ASHH sign, Anvil-shaped lesion, Secondary CNV

## Abstract

To evaluate the efficacy of swept-source optical coherence tomography (SS-OCT) in detecting the clinical spectrum of macular microstructure changes secondary to laser pointer injury (LPI), including their response to therapeutic intervention. Methods: A retrospective study, including consecutive patients with LPI. Inclusion criteria were visual symptoms and biomicroscopic, OCT, and fundus fluorescein angiography (FFA) features of LPI. We used the SS-OCT for imaging and the swept-source optical coherence tomography angiography (SS-OCTA) to confirm or exclude the diagnosis of a choroidal neovascular membrane (CNV). We used FFA to confirm the diagnosis of a CNV whenever SS-OCT and SS-OCTA images were insufficient to establish its presence. The outcome measures were the morphological features in the macula secondary to LPI and the response of CNV to aflibercept. Results: The study included 31 eyes of 22 patients. Ten patients (45%) were ≤ 15 years old. Laser pointer maculopathy (LPM) was bilateral in 9 patients (41%). The mode of injury was self-inflicted in 14 patients (64%). Central scotoma was the most common symptom reported by the patients. The mean baseline best-corrected visual acuity (BCVA) was 20/50. The mean follow-up period was 9.6 months. The mean final BCVA was 20/40. Acute stages of LPM were characterized by focal or diffuse disruption of the outer retinal layers, subretinal hyperreflective mound, anvil-shaped lesion, or the angular sign of Henle fiber layer hyperreflectivity (ASHH). The features of chronic stages included secondary CNV or macular holes. CNV and macular hole were common features in both acute and chronic stages. The most common LPI-induced macular lesion was retinal pigment epithelium (RPE) changes. Type II CNV developed in three eyes (10%). Conclusion: SS-OCT depicted a characteristic morphological profile of LPM in the acute and chronic stages. SS-OCTA is a non-invasive and reproducible complementary tool in detecting secondary CNV and monitoring its response to therapy.

## Background

In recent years, there has been a surge in the number of individuals sustaining macular injuries due to exposure to handheld laser pointers [1, 2]. The mode of exposure is accidental, deliberate, or even self-inflicted [1–3]. Several factors come in tandem to perpetuate the occurrence of macular injury caused by laser pointers. The ease of access to these devices, mislabeling of laser class and laser output, and public unawareness of their detrimental risks to the eye [1–3]. Furthermore, delayed diagnosis is common as children who constitute the majority of cases frequently refrain from reporting playing with laser pointers to avoid punishment [2, 4]. Notably, ophthalmologists often confuse the condition with similar macular diseases, especially macular dystrophies [2–4]. Laser pointers emit long and short ranges of laser wavelengths, including red and green lasers. The human retina will sustain damage when viewing a Class 3 A laser device emitting an output power of 5 mW for 10 s. [5, 6] The severity of damage will depend on the laser wavelength, the laser output power, the proximity of the laser beam to the eye, the clarity of ocular media, the amplification of retinal laser irradiance by the ocular condensing structures, the degree of fundus pigmentation, and the natural protective mechanisms such as the aversion reflex, blinking reflex, and pupil constriction [7–10]. Ultra-high-resolution optical coherence tomography (UHR-OCT) enabled an unprecedented in vivo visualization of the microstructure of the retinal layers. It represented a breakthrough in detecting the pathological changes in the macula secondary to LPI and studying their temporal evolution from acute through chronic stages, hence establishing a disease pattern that helped distinguish LPM from other macular diseases [1, 2, 4, 11, 12]. The present study aims to evaluate the efficacy of SS-OCT in detecting the clinical spectrum of macular microstructure changes secondary to LPI, including their response to therapeutic intervention.

## Patients and methods

This retrospective case series includes all consecutive patients who presented to a retina tertiary center with a history of LPI between 2015 and 2024. The patients presented in person seeking medical advice or were referred by other ophthalmologists. The study included patients who complained of unilateral or bilateral visual symptoms such as scotoma, blurred vision, or metamorphopsia, whose examination revealed a macular lesion and gave a history of exposure to laser-emitting devices, including laser pointers or toys. We defined LPM on biomicroscopy during the acute phase as a yellowish or reddish-yellow sharply delineated sub-retinal lesion in the foveal or juxta-foveal area. Other associated features include submacular hemorrhage, retinal pigmentary changes, or CNV. In the chronic phase, biomicroscopy features include macular atrophy, scarring, or secondary complications such as a macular hole. By OCT examination, we defined LPM during the acute phase as one or more of the following features: (i) Focal or diffuse disruption of the outer retinal layers that involves the inner segment/outer segment (IS/OS) photoreceptor layer. These defects could be solitary or multiple and located in the subfoveal (ellipsoid zone), or extrafoveal area. (ii) Subretinal hyperreflective mound in the subfoveal, or extrafoveal area and might be associated with disruption of Bruch’s membrane. (iii) Curvilinear hyperreflective bands originate at the RPE and extend to the outer plexiform layer (OPL). These bands follow the course of the Henle fiber layer (HFL). These lesions are the anvil-shaped lesion [1] or the ASHH sign [13]. OCT features of chronic LPM include secondary CNV, or macular holes that could be full-thickness or lamellar, extending from the RPE to the external limiting membrane (ELM). The hole has characteristic rectangular straight edges and is lined by a hyperreflective material along the top and the edges of the hole. Secondary CNV and macular holes were considered a common feature of acute and chronic injuries, depending on the intensity of the laser, the degree of burn, and the consequent disruption of the retinal layers and the Bruch’s membrane. The study excluded all patients with macular dystrophies, a history of macular disease secondary to ocular trauma, uveitis, retinal vascular disease, intraocular infection, myopic maculopathy, or drug-induced maculopathy. All recruited patients had a full ophthalmological examination. This included assessment of BCVA using Snellen acuity chart and later converted to decimal units for descriptive statistics, intra-ocular pressure (IOP) measurement using Goldmann’s applanation tonometry or air-puff tonometer, slit-lamp examination including fundus biomicroscopy, and indirect ophthalmoscopy. We performed the OCT examination using the SS-OCT; DRI OCT Triton machine version 10.11 (Topcon Corporation, Tokyo, Japan). The software incorporates SS-OCTA and OCTARA (Optical Coherence Tomography Angiography Ratio Analysis). The scanning protocol for the macular area consisted of a radial scan that deployed 12 lines centered on the fovea, each line is 9 mm; 1024 A-scans, a thickness map, and a high-definition line scan for high-resolution lesion evaluation. We defined CNV on SS-OCT images as any one or more of the following signs: Fusiform or nodular hyper-reflective lesion related to the RPE with associated sub- and/or intra-retinal or sub-RPE fluid or hyperreflective amorphous material, pitchfork sign, hemorrhage, and focal RPE disruption. We performed SS-OCTA whenever SS-OCT revealed signs suggestive of a CNV or was inconclusive in excluding CNV. We performed FFA using a Topcon TRC 50DX fundus camera (Topcon Corporation, Tokyo, Japan). We reserved the use of FFA for patients whose SS-OCT and SS-OCTA images were inconclusive for CNV presence due to the invasive nature of FFA, since many patients in the study were children. The main outcome measure was the evaluation of the morphological features in the macula secondary to LPI. The secondary outcome measure was the evaluation of the response of CNV to intravitreal injection of aflibercept.

## Results

The study included 31 eyes of 22 patients. Seventeen patients (77%) were males. The mean age was 18 years (range: 11–45; SD 8). Ten patients (45%) were ≤ 15 years old. LPI maculopathy was bilateral in 9 patients (41%). The mode of injury was self-inflicted in 14 patients (64%). Self-inflicted LPI was seen particularly in children and teenagers due to curiosity or staring intentionally at a beam during peer challenge. Central scotoma was the most commonly reported symptom among patients. It occurred in 19 eyes (61%). The remaining patients presented with blurred vision in the affected eye (12 eyes; 39%). The mean baseline BCVA was 20/50 (0.4) (range: 20/400 (0.05)–20/20 (1); SD 0.3). Seventeen eyes (55%) had BCVA < 20/40 (0.5) at presentation. The mean follow-up period was 9.6 months (range: 1–56; SD 16.6). The mean final BCVA was 20/40 (0.5) (range: 20/400 (0.05)–20/20 (1); SD 0.3). Seventeen eyes (55%) had BCVA ≥ 20/40 (0.5) at the last follow-up visit. The most common LPI-induced macular lesion was the RPE changes, such as mottling, pigment clumps formation, and atrophy. These changes were detected in 21 eyes (68%), followed by outer retinal layer defect/hole formation in 17 eyes (55%). In OCT images, five eyes (16%) had perifoveal or parafoveal anvil-lesion or the ASHH sign. Secondary CNV located in the sub-retinal space above the RPE layer (type II), sub-macular hemorrhage, and sub-macular scarring developed in three eyes each (10%). Two eyes (6%) had a yellowish or reddish-yellow submacular lesion on biomicroscopic examination. (Table 1).

**Table 1:** Baseline patients’ characteristics

Baseline characteristics	N (%)
Gender	
Male	17 (77)
Female	5 (23)
Age, years	
< 15	8 (36)
15–25	12 (54.5)
> 25	2 (9)
Mode of LPI (per patient)	
Self-inflicted	14 (64)
Accidental	8 (36)
Laterality (per patient)	
OD	7 (32)
OS	6 (27)
OU	9 (41)
Symptoms (per eye involved)	
Central scotoma	19 (61)
Blurred vision	12 (39)
Follow-up period in months (per eye involved)	
< 6	16 (52)
6–12	0 (0)
> 12	6 (19)
Mean baseline BCVA	20/50 (0.4)
< 20/63 (0.3)	12 (39)
20/63 (0.3)–20/40 (0.5)	10 (32)
> 20/40 (0.5)	9 (29)
LPI maculopathy feature (per eye involved)	
Biomicroscopic examination	
RPE changes	21 (68)
Yellowish/reddish-yellow submacular lesion	2 (6)
SS-OCT examination	
Outer retinal layer defect/hole	17 (55)
Anvil lesion/ASHH sign	5 (16)
CNV	3 (10)
Submacular hemorrhage	3 (10)
Submacular scarring	3 (10)

ASHH; angular sign of Henle fiber layer hyperreflectivity, BCVA, best-corrected visual acuity; CNV, choroidal neovascularization; LPI, laser pointer injury; OD, oculus dexter; OS, oculus sinister; OU, oculus uterque; RPE, retinal pigment epithelium

### Case presentation

**Fig. 1:**
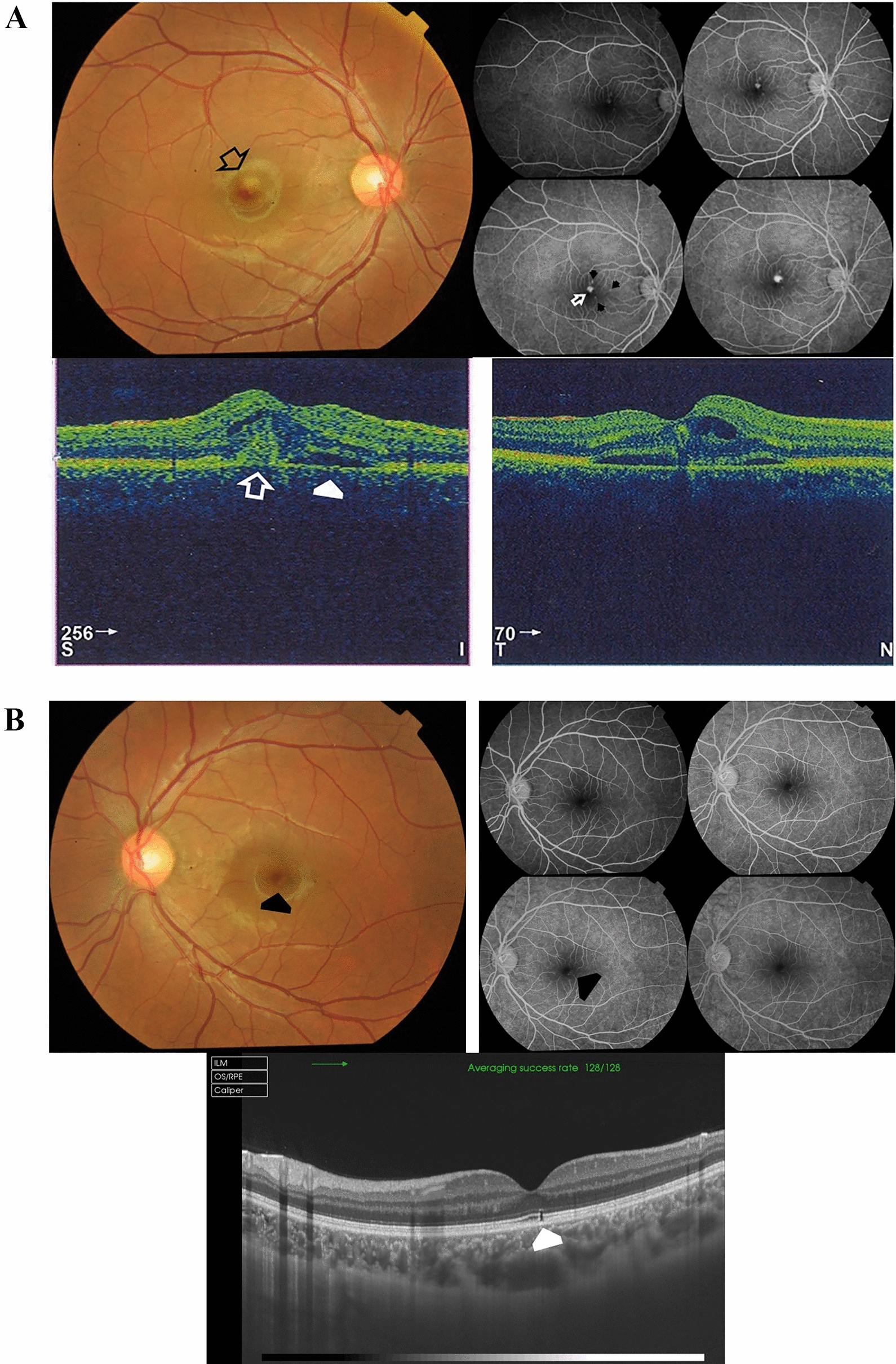

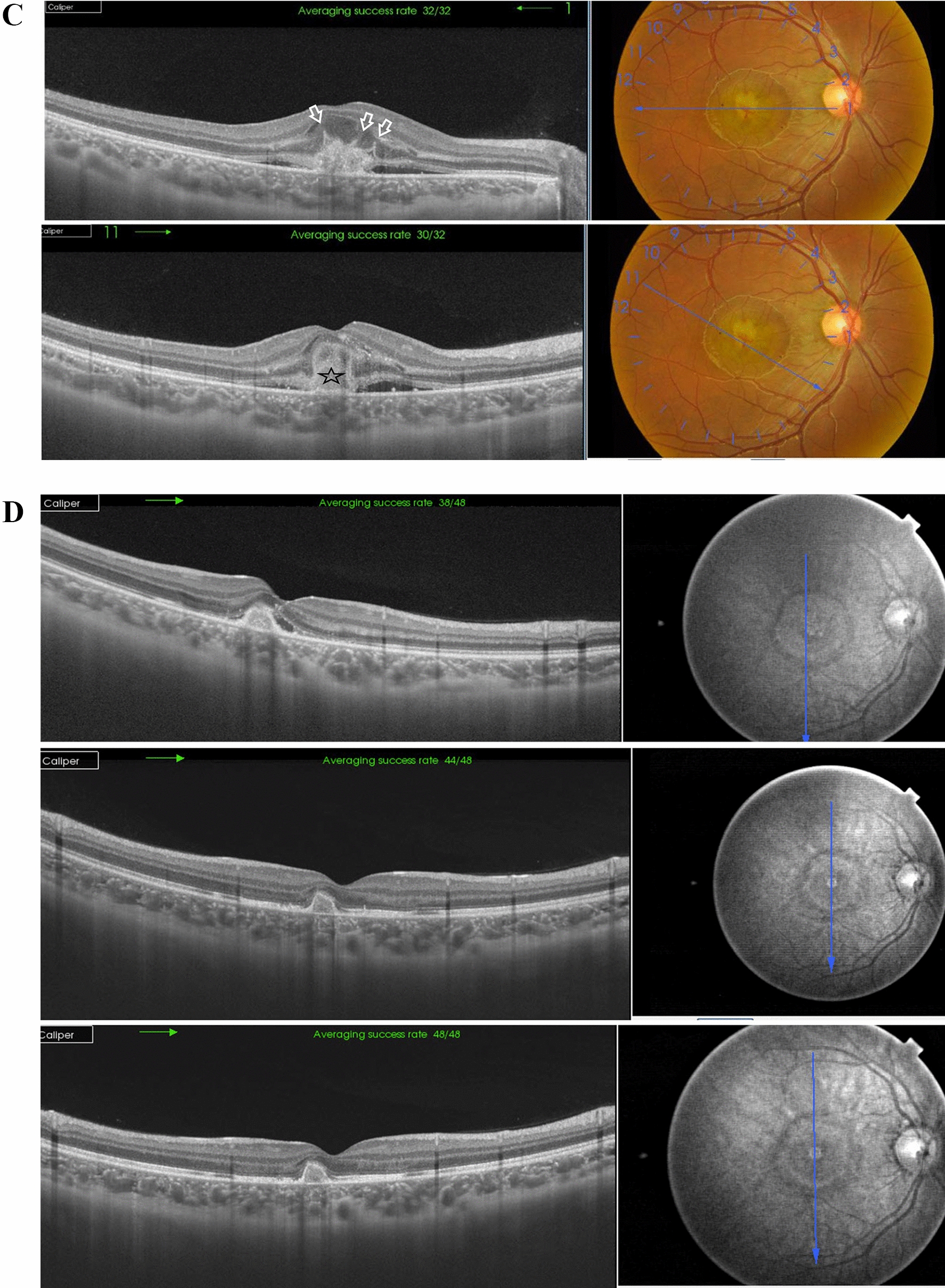

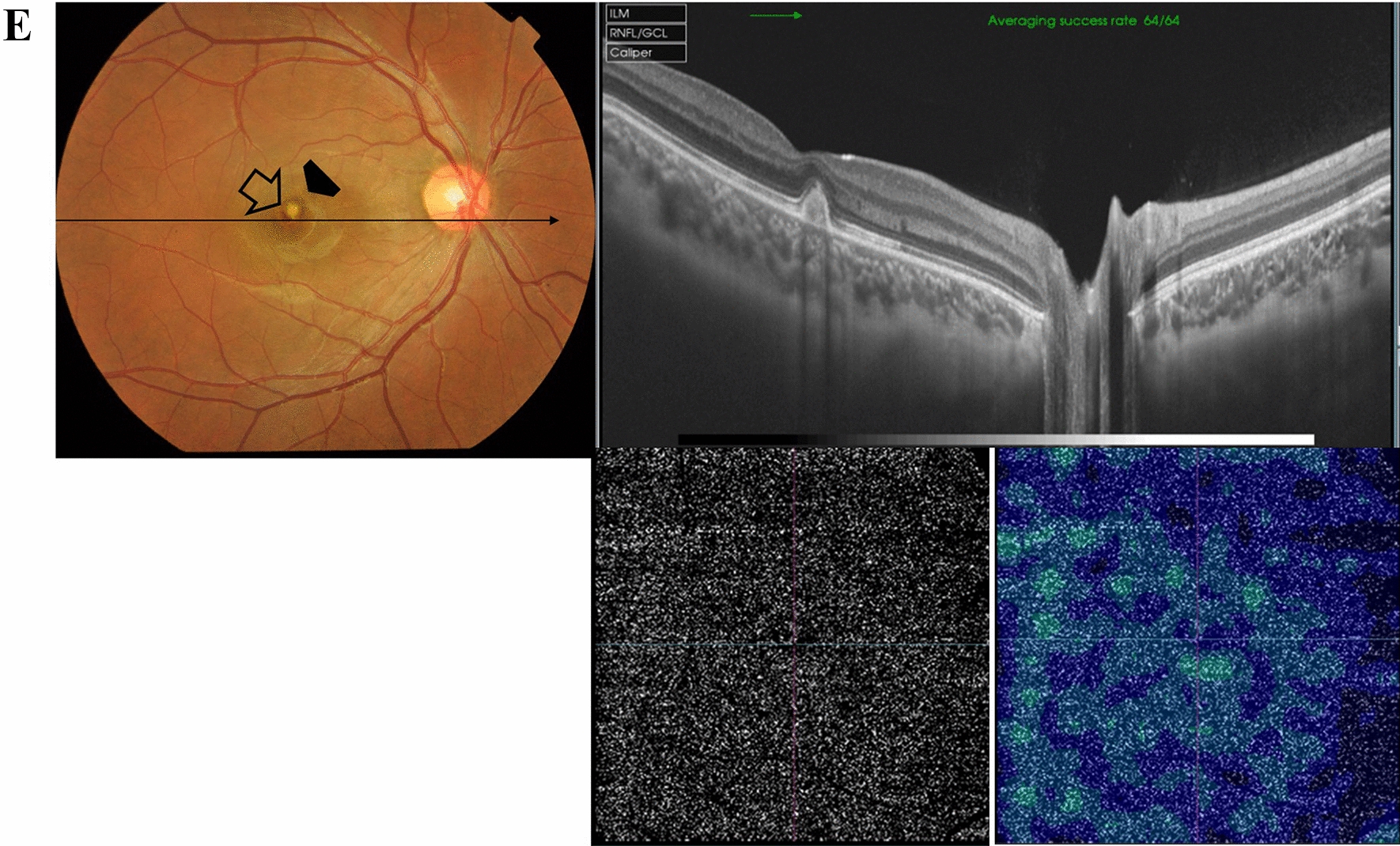
**A** Top left. Color fundus photo of the right eye of an 11-year-old boy shows a subfoveal yellowish lesion reminiscent of an acute laser burn with macular edema and a lost foveal reflex (black arrow). Top right. In fundus fluorescein angiography (FFA), the subfoveal lesion shows early hyperfluorescence that increased in size and intensity, indicating an active subfoveal choroidal neovascularization (CNV), white arrow. Note that the subfoveal lesion is surrounded by smaller hyperfluorescent spots due to faint staining of the light laser burns (black arrows). Bottom left and right. Optical coherence tomography (OCT) images of the right macula show a hyperreflective amorphous lesion lying above the retinal pigment epithelium (RPE) with an overlying cuff of fluid (open white arrow), diffuse spongy edema of the macula, and an associated focal neurosensory detachment (white arrowhead), indicating type II CNV. **B** Top left. Color fundus photo of the left eye shows mild RPE mottling in the macular area (black arrowhead). Top right. The corresponding FFA shows a hyperfluorescent spot corresponding to the laser burn (black arrowhead). Bottom. Swept-source optical coherence tomography (SS-OCT) image in a line scan mode shows a focal defect in the IS/OS photoreceptors layer and the cone outer segment termination line (white arrowhead). **C** Top and bottom images, one week from the first presentation. SS-OCT images of the right macula in a radial scan mode show a type II CNV with a surrounding cuff of sub-retinal fluid. Note the pitchfork sign (white arrows) and the multiple intraretinal hyperreflective foci suggesting the inflammatory nature of the CNV. The non-uniform hyperreflectivity of the lesion corresponds to the deep retinal hemorrhage seen in the color photo (black asterisk). **D** Top. SS-OCT images of the right macula in a line scan mode, one month after the patient received the first aflibercept intravitreal injection. Middle and Bottom. SS-OCT images of the right macula in a line scan mode, one and two months after the patient received the second aflibercept intravitreal injection, respectively. Note the resolution of edema and regression of the CNV. **E** Top left. One year later, the color fundus photo of the right eye shows a persistent subfoveal yellowish lesion (open black arrow) and surrounding focal RPE mottling (black arrowhead). Top right. SS-OCT image in a high-definition line scan mode shows resolution of the previously noted focal neurosensory detachment and sub-retinal fluid with persistent knob-like subfoveal hyperreflective lesion. Bottom photos. Swept-source optical coherence tomography angiography (SS-OCTA) images in a 6 × 6 mm field of the outer retinal layer and the corresponding flow density map show a normal homogenous hypointense signal

Case # 1. An 11-year-old boy presented to our clinic complaining of a stationary dark spot in front of his right eye for one week. His parents mentioned that he received a laser pointer as a gift. Examination revealed a well-demarcated subfoveal yellowish lesion. His BCVA in the right eye was 20/200, and 20/20 in the left eye. The fundus of the left eye showed mild RPE changes in the macula. FFA and OCT images of the right eye revealed an active type II CNV (Fig. 1A). OCT images of the left eye revealed a subfoveal focal disruption of the ellipsoid zone (Fig. 1B). We recommended intravitreal aflibercept injection for the right eye, but the parents went to seek another opinion, only to return one week later with the child complaining of a marked drop in vision in the right eye. His BCVA in the right eye was counting fingers (CF) at 1 m (Fig. 1C). The patient received two aflibercept injections that resulted in complete regression of the CNV. His BCVA improved to 20/25 (Fig. 1D). The patient remained stable over the ensuing 1 year of follow-up (Fig. 1E).

**Fig. 2:**
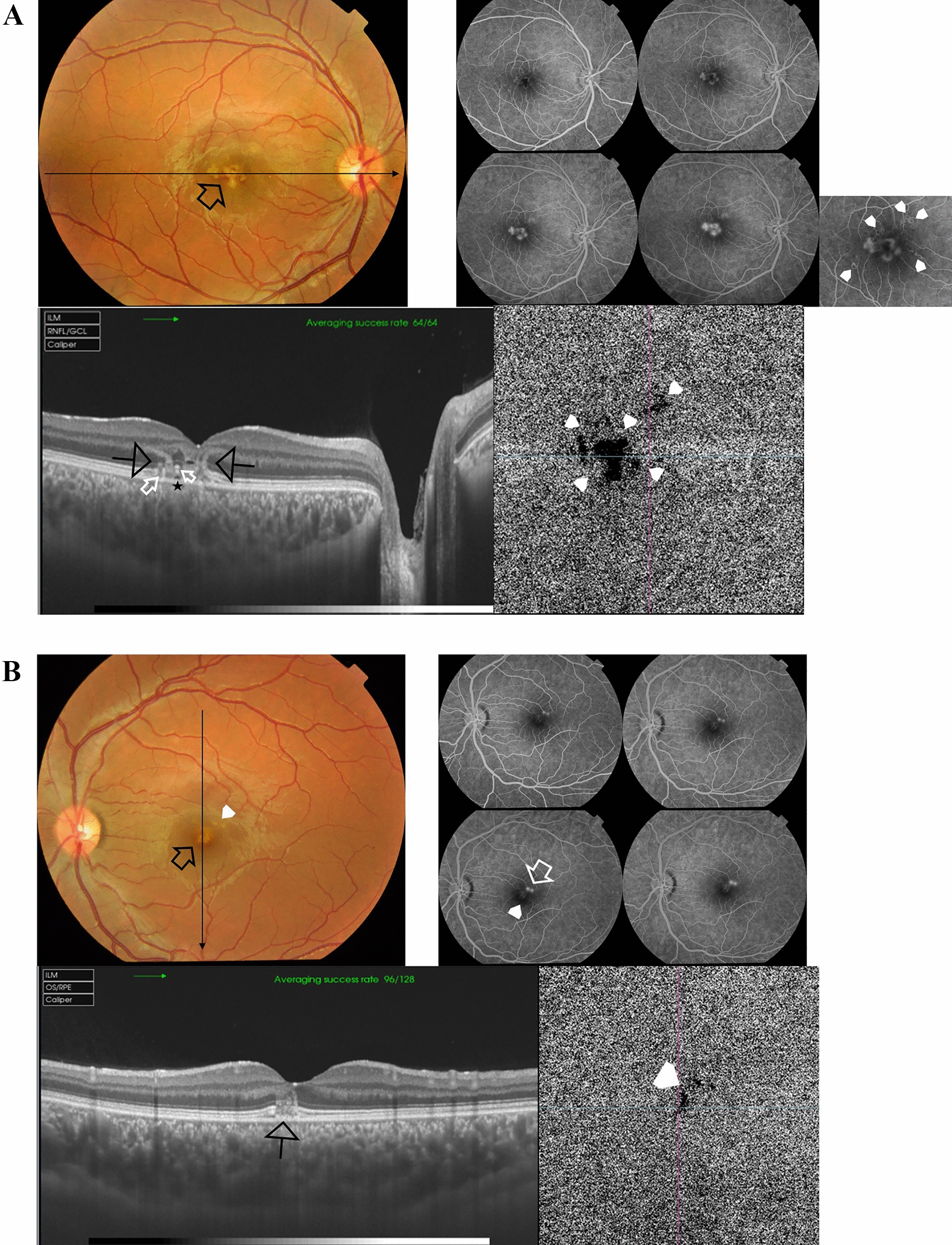

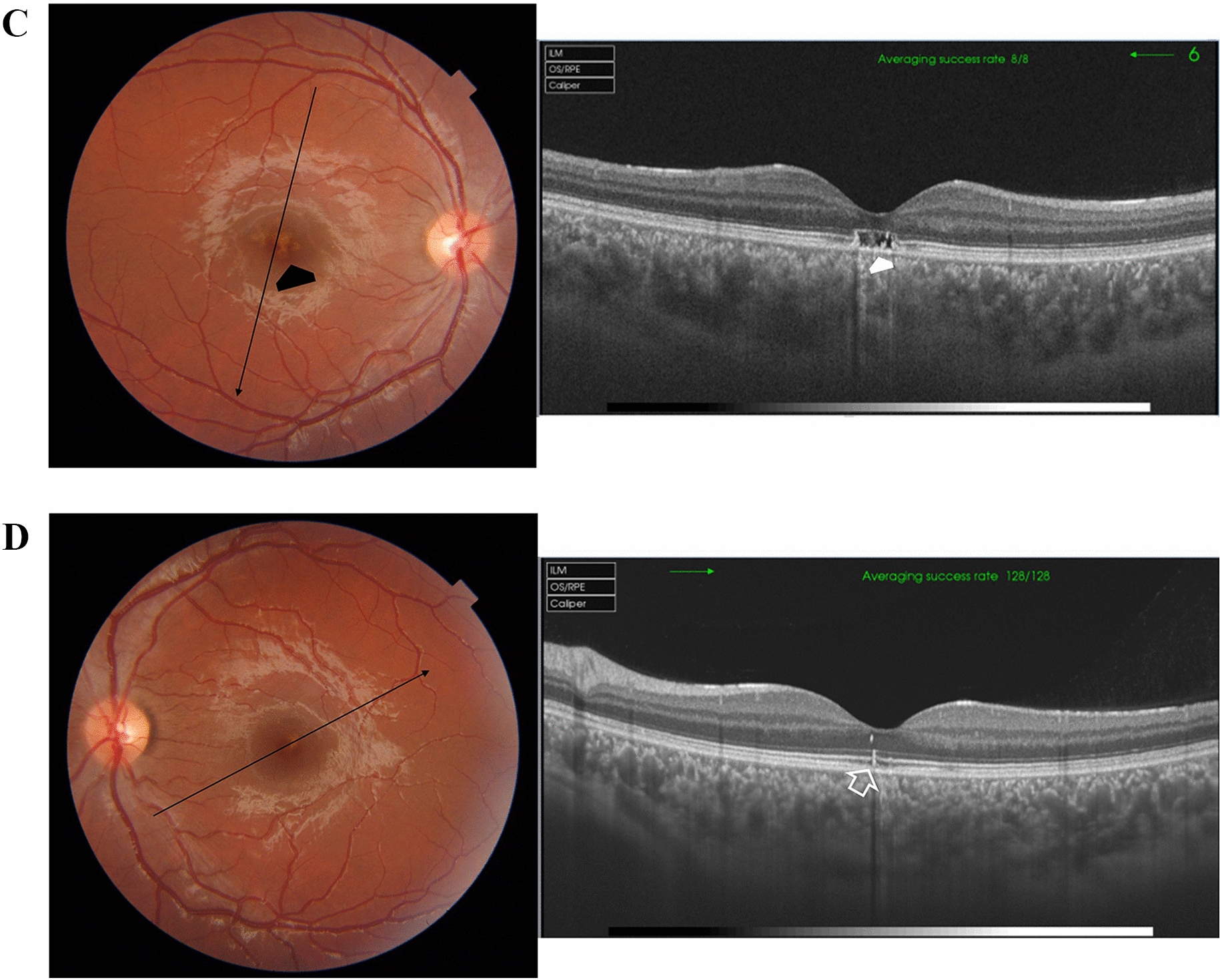
**A** Top left. Color fundus photo of the right eye of a 13-year-old boy shows subfoveal yellowish-white multiple lesions arranged in a cluster pattern (black arrow) reminiscent of acute laser burns. Top right. In fundus fluorescein angiography (FFA), the subfoveal lesion shows an early hyperfluorescent rim and a central blocked fluorescence, denoting retinal pigment epithelium (RPE) clumps formation. In late frames, there are variable degrees of staining of the lesion. The subfoveal lesion is surrounded by multiple scattered foci of central blocked fluorescence representing RPE hyperplasia and a surrounding hyperfluorescent rim (inset; white arrowheads). Bottom left. Swept-source optical coherence tomography (SS-OCT) image of the right macula in a high-definition line scan mode shows the perifoveal angular sign of Henle fiber layer hyperreflectivity (ASHH) sign/anvil-shaped lesion due to enhanced visualization of the Henle fiber layer (black arrows). There are hyperreflective dots (white arrows) in the outer retina reminiscent of migrating RPE. These dots correspond to the central blocked fluorescence and late staining in the FFA image. Note the hyporeflective space (asterisk) indicating focal disruption of the outer retinal layers by the laser energy. Bottom right. Swept-source optical coherence tomography angiography (SS-OCTA) image in a 6 × 6 mm field of the choriocapillaris shows focal areas of rarefaction (white arrowheads) corresponding to the subfoveal lesion and denoting bidirectional dissipation of laser energy absorbed by melanin in the RPE into the outer and inner retina and the underlying choriocapillaris. **B** Top left. Color fundus photo of the left eye shows a subfoveal yellowish-white irregular lesion (black arrow) and two small subretinal foci of RPE mottling at the temporal edge of the fovea (white arrowhead). Top right. In FFA, the subfoveal lesion shows early hyperfluorescence (white arrowhead) and late staining of the other two foci of RPE mottling (white arrow). Bottom left. SS-OCT image of the left macula in a line scan mode shows a perifoveal ASHH sign (black arrow). Bottom right. SS-OCTA image in a 6 × 6 mm field of the choriocapillaris shows focal rarefaction (white arrowhead) corresponding to the subfoveal lesion. **C** Left. Color fundus photo of the right eye, one month later. Note the subfoveal RPE mottling (black arrowhead). Right. SS-OCT image of the right macula in a radial scan mode shows the development of an outer retinal cavitation and loss of outer retinal layers with characteristic sharp rectangular edges extending from the RPE to the external limiting membrane (ELM). Note the hyperreflective ring surrounding the top and the edges of the outer cavitation representing cellular debris (white arrowhead). **D** Left. Color fundus photo of the left eye, one month later. Note the RPE mottling in the foveal area. Right. SS-OCT image of the left macula in a line scan mode shows complete resolution of the previously noted ASHH sign, and complete restoration of the ellipsoid zone and the outer retinal layers. Note the residual interrupted vertical candle-shaped hyperreflective streak (white arrow) extending from the RPE to the outer plexiform layer (OPL) along the border of the resolved ASHH sign

Case # 2. A 13-year-old boy was referred by another ophthalmologist for a fluorescein angiogram for a bilateral macular lesion. The referring ophthalmologist made a provisional diagnosis of macular dystrophy. The parents reported that their son complained of blurred vision 2 weeks ago following a tolerance challenge with a friend, in which they mutually shone a laser pointer in each other’s eyes. The BCVA was 20/200 in the right eye and 20/80 in the left eye. Fundus examination revealed multiple bilateral subfoveal yellowish-white lesions. OCT revealed the anvil-shaped lesion/ASHH sign in the macula of both eyes, with a more severe presentation in the right eye (Fig. 2A and B). One month later, the macular lesion in the right eye progressed to an outer retinal hole, and the left eye lesion showed almost complete resolution (Fig. 2C and D). At this stage, the BCVA in the right eye improved to 20/80 and the left eye improved to 20/25.

**Fig. 3:**
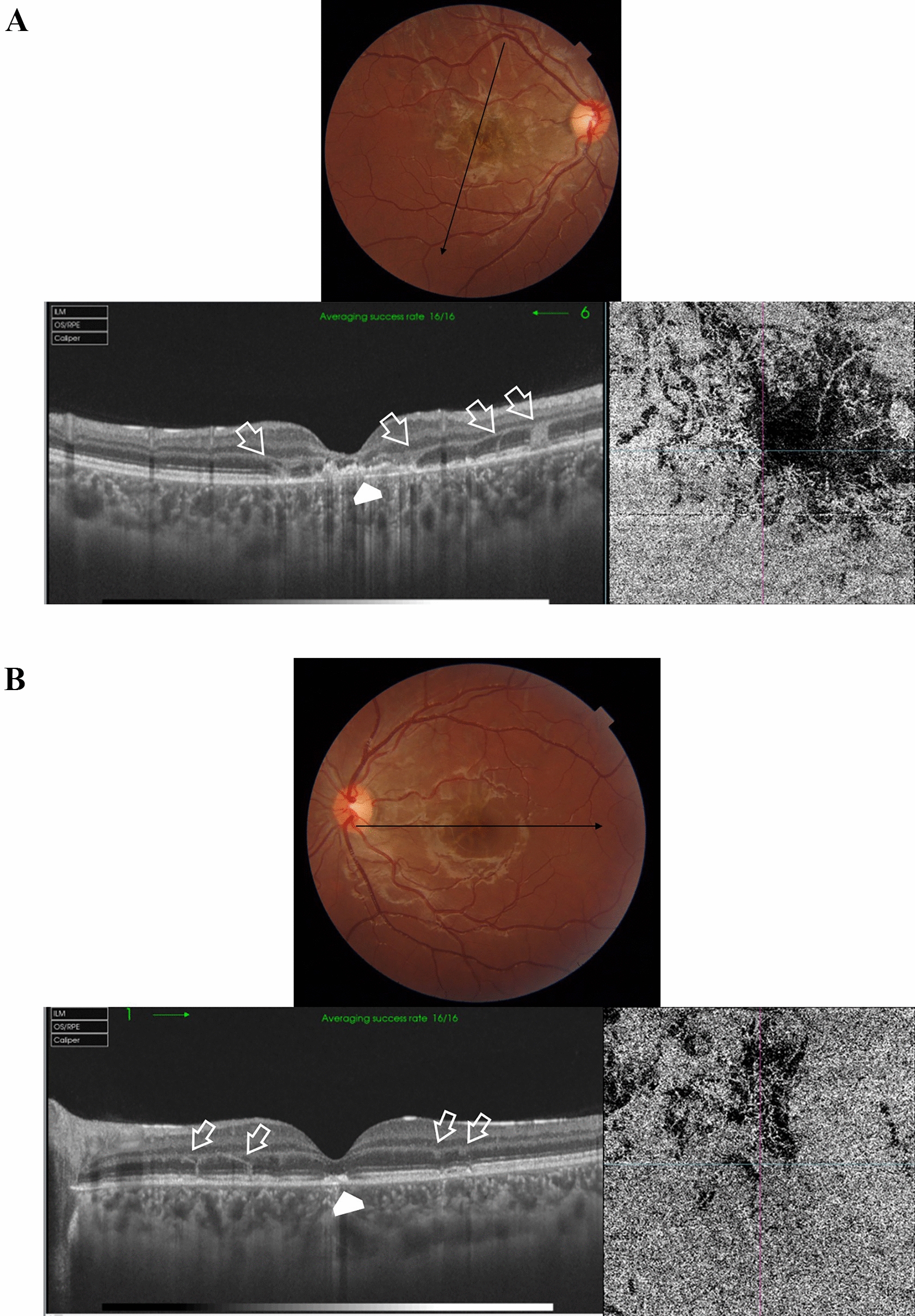
**A** Top. Color fundus photo of the right eye of a 15-year-old girl shows diffuse subfoveal retinal pigment epithelium (RPE) mottling. Bottom left. Swept-source optical coherence tomography (SS-OCT) image of the right macula in a radial scan mode shows multiple parafoveal angular sign of Henle fiber layer hyperreflectivity (ASHH) (white arrows). Note the subfoveal irregular and thickened RPE (white arrowhead). Bottom right. Swept-source optical coherence tomography angiography (SS-OCTA) image in a 4.5 × 4.5 mm field of the choriocapillaris showing multifocal rarefaction indicating bidirectional dissipation of laser energy from the melanin of the RPE inwards to the outer and inner retina and outwards to the choriocapillaris. **B** Top. Color fundus photo of the left eye shows diffuse subfoveal RPE mottling. Bottom left. SS-OCT image of the left macula in a radial scan mode shows multiple parafoveal ASHH signs (white arrows). Note the subfoveal irregular and thickened RPE (white arrowhead). Bottom right. SS-OCTA image in a 4.5 × 4.5 mm field of the choriocapillaris showing multifocal rarefaction

Case # 3. A 15-year-old girl presented to our clinic complaining of blurred vision in both eyes 2 weeks ago when she accidentally viewed a laser beam shining from a device during a wedding party. The BCVA was 20/80 in both eyes. Fundus examination revealed bilateral diffuse RPE mottling in the macular area. OCT revealed the ASHH sign in multiple parafoveal locations in both eyes (Fig. 3A, B).

**Fig. 4:**
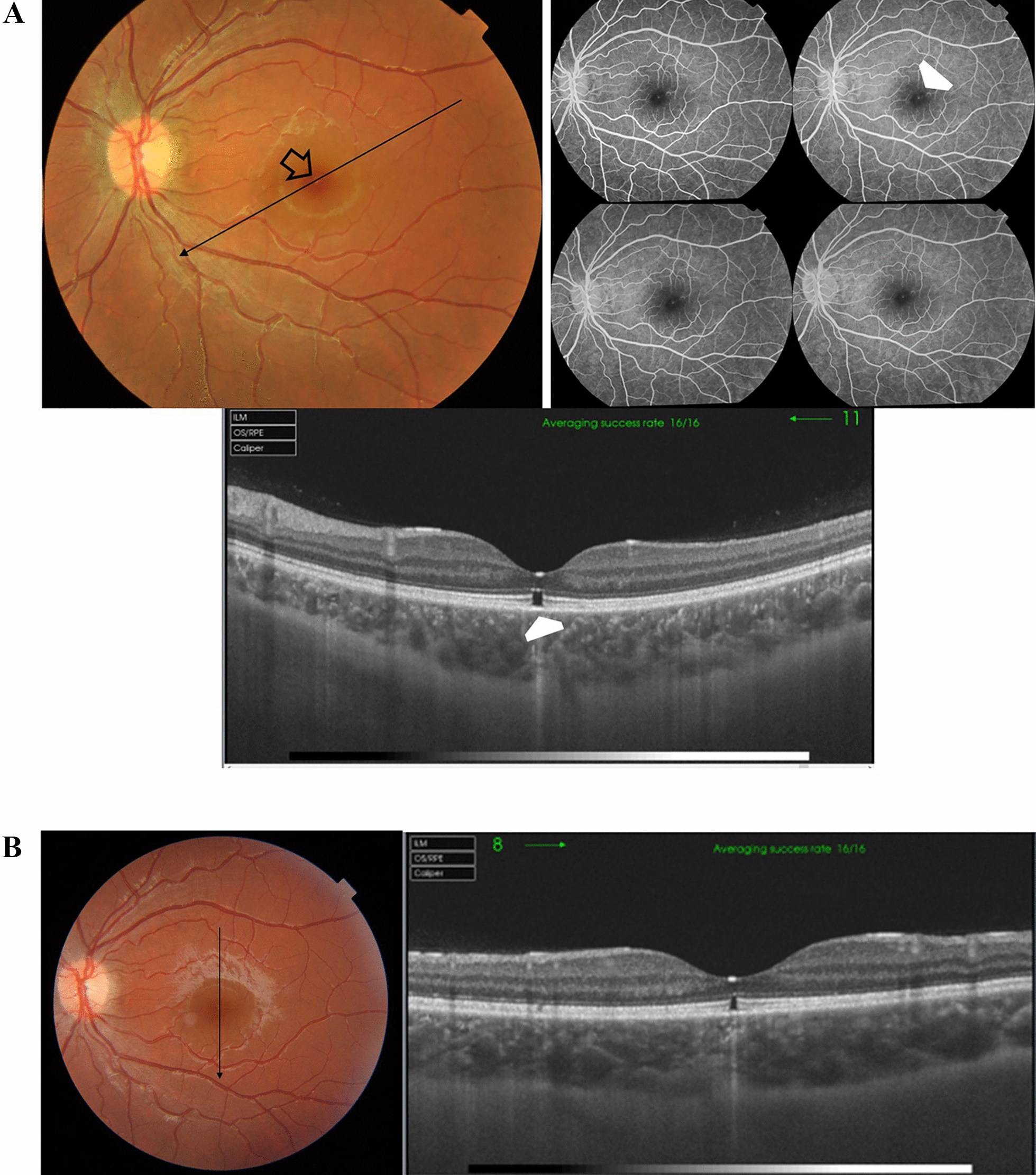
**A** Top left. Color fundus photo of the left eye of a 14-year-old girl shows subfoveal retinal pigment epithelium (RPE) mottling (black arrow). Top right. In fundus fluorescein angiography (FFA), the subfoveal lesion shows focal hyperfluorescence corresponding to the laser burn site (white arrowhead). Bottom. Swept-source optical coherence tomography (SS-OCT) image of the left macula in a radial scan mode shows an outer retinal hole (white arrowhead). **B** Left. Color fundus photo of the left eye after 9 months shows stationary subfoveal RPE mottling. Right. SS-OCT of the left macula in a 5-line raster scan mode shows a mild reduction in the size of the outer retinal hole

Case # 4. A 14-year-old girl presented to our clinic complaining of blurred vision in the left eye for 10 days after accidentally viewing a laser pointer shone by her classmate. The BCVA was 20/63. Fundus examination of the left eye revealed a subtle RPE mottling. Her right eye was normal with a BCVA of 20/20. OCT examination of the left eye showed an outer retinal hole. Nine months later, the outer retinal hole diminished in size mildly. BCVA remained 20/63. (Fig. 4A, B).

**Fig. 5:**
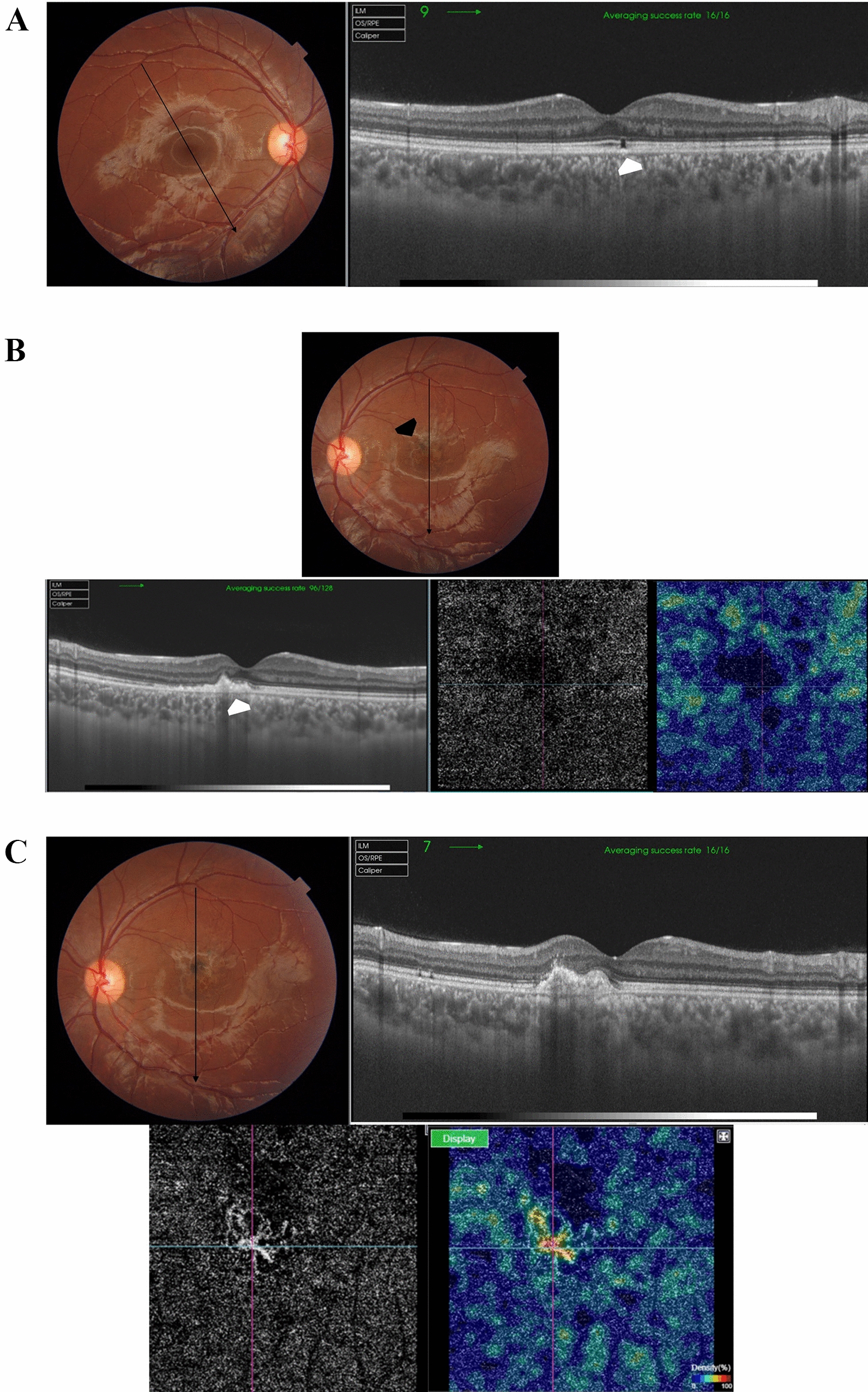
**A** Left. Color fundus photo of the right eye of a 14-year-old boy shows mild subfoveal retinal pigment epithelium (RPE) mottling. Right. Swept-source optical coherence tomography (SS-OCT) image of the right macula in a radial scan mode shows an outer retinal hole (white arrowhead). **B** Top. Color fundus photo of the left eye shows extensive subfoveal RPE changes. Note the RPE pigment clump formation (black arrowhead). Bottom left. SS-OCT image of the left macula in a line scan mode shows a subfoveal hyperreflective amorphous lesion (white arrowhead). Bottom middle, and right photos. Swept-source optical coherence tomography angiography (SS-OCTA) images in a 3 × 3 mm field of the outer retina and the corresponding flow density map show a normal homogenous hypointense signal of the outer retina. **C** Top left and top right. Two years later the color fundus photo and the SS-OCT image (radial scan mode) invariably revealed no change compared to the previous visit, however, the corresponding SS-OCTA images (3 × 3 mm field) of the outer retina and the corresponding color-coded flow density map (bottom left and right) show an inactive neovascular membrane and a residual flow in a vascularized scar

Case # 5. A 14-year-old boy presented to our clinic. His parents mentioned that he was enrolled in a vision screening campaign in school, where they discovered defective vision in both eyes. His BCVA in the right eye was 20/40, and in the left eye was 20/200. His fundus examination revealed mild subfoveal RPE mottling in the right eye and extensive subfoveal RPE changes, including mottling and hyperplasia, in the left eye. OCT examination revealed an outer retinal hole in the right eye and a subfoveal amorphous hyperreflective lesion in the left eye that corresponded to the subfoveal RPE hyperplasia in the color photo. OCTA did not reveal evidence of choroidal neovascularization (Fig. 5A and B). Two years later, the child returned complaining of a recent drop of vision in the left eye. His BCVA in the left eye was 20/200. OCT revealed the same picture as the last visit. However, OCTA revealed a vascularized scarred type II CNV. His BCVA remained 20/200 over the following 3 years (Fig. 5C).

**Fig. 6:**
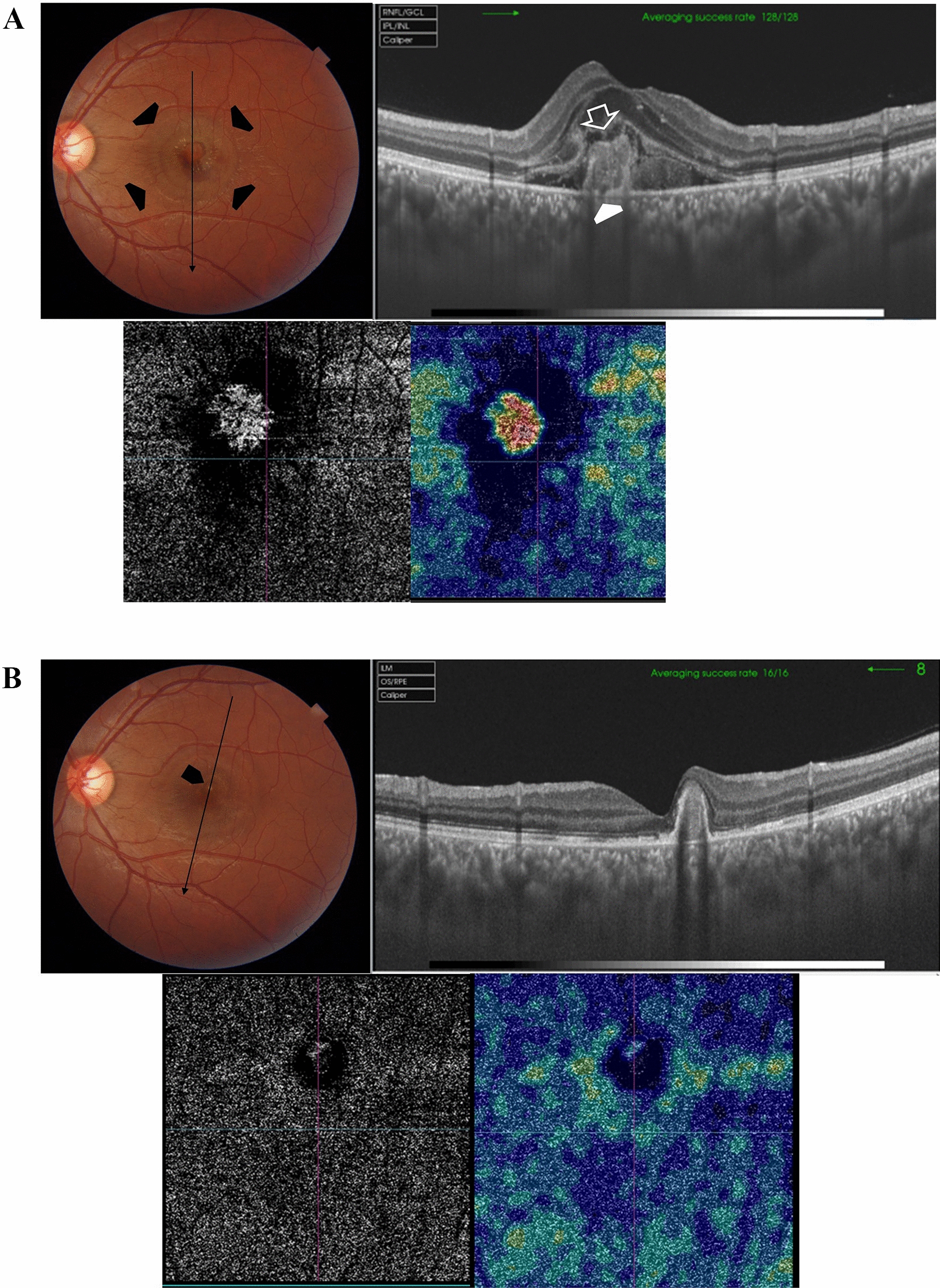
**A** Top left. Color fundus photo of the left eye of a 19-year-old male patient shows a circumscribed area of macular neurosensory detachment and submacular hemorrhage (black arrowheads). Top right. Swept-source optical coherence tomography (SS-OCT) image of the left macula in a line scan mode shows a subfoveal amorphous hyperreflective lesion and an adjacent hyporeflective mound. Note the surrounding cuff of sub-retinal fluid and overlying intra-retinal cystic spaces (white arrow). Note the focal subfoveal hyporeflective defect in the retinal pigment epithelium (RPE) layer (white arrowhead), reminiscent of a focal disruption by the laser impact. Bottom left and right. Swept-source optical coherence tomography angiography (SS-OCTA) images in a 3 × 3 mm field of the outer retina and the corresponding flow density map show an active neovascular network with dense vascular arborization, looping, and anastomosis. **B** Top left. Color fundus photo of the left eye 6 months after a single intravitreal aflibercept injection. Note the resolution of the previously noted neurosensory detachment and submacular hemorrhage and the development of a circumscribed yellowish submacular lesion (black arrowhead). Top right. SS-OCT image of the left macula in a radial scan mode shows a subfoveal knob-like hyperreflective lesion with complete resolution of the previously noted neurosensory detachment. Note the attenuation of the subfoveal ellipsoid zone. Bottom. SS-OCTA images in a 3 × 3 mm field of the outer retina and the corresponding flow density map show almost complete regression of the previously noted active CNV with a minimal flow in the neovascular frond

Case # 6. A 19-year-old male patient presented to our clinic complaining of a marked decrease in vision in his left eye. He gave a history of an already diminished vision in the same eye after intentionally shining a laser pointer in his left eye 2 weeks ago, but it was aggravated for 3 days. His BCVA was 20/200. His fundus examination revealed a circumscribed area of macular neurosensory detachment and submacular hemorrhage. OCTA examination revealed an active CNV (Fig. 6A). He received a single intravitreal aflibercept injection that resulted in regression of the CNV with a residual juxta-foveal organized scar. His BCVA improved to 20/25. His vision remained stable, and he had no recurrences over the following 6 months (Fig. 6B).

## Discussion

In the present study, the predominant involvement of outer retinal layers and the choroid by LPM is in the form of RPE changes, outer retinal holes, and anvil-shaped lesions. Secondary type II CNV developed in three eyes. This pathological profile is determined by the laser tissue interaction that develops upon the impact of the laser and the ocular tissues [1]. The RPE has a high concentration of melanin, an avid absorber of laser energy. The energy absorbed by melanin is converted into heat that produces photothermal damage due to denaturation of proteins, cell disruption, and secondary inflammation [3, 14]. Thermal damage produces an array of lesions in the RPE layer, ranging from mild pigmentary changes to atrophy and necrosis [5, 15, 16]. Further thermal energy dissipation to the HFL causes opacification and thickening due to inflammation, with consequent disruption of the normal arrangement of its fibers. This changes the optical properties of the HFL and renders it more reflective on OCT, producing hyperreflective curvilinear bands extending from the RPE to the OPL known as the anvil-shaped or ASHH sign. Additionally, thermal energy dissipation could produce outer retinal holes [1, 11–13]. Short-wavelength lasers have high energy and less penetration relative to longer-wavelength lasers, and they are absorbed preferentially by melanin in the RPE. Therefore, they are more likely to damage the retinal layers. In addition to photothermal damage, laser energy produces a photochemical reaction that consists of nonthermal damage caused by triggering a biochemical reaction that enhances the production of toxic free radicals and the resultant apoptosis of RPE cells and the photoreceptors. The final visual acuity of the patient will depend on the degree of damage inflicted on the photoreceptors [1–3, 9, 10, 14, 17, 18]. Another important cause of vision compromise is the development of a secondary CNV due to focal disruption of Bruch’s membrane by the acute inflammation caused by laser energy [3, 5]. The macular lesions produced by laser pointers frequently demonstrate peculiar morphology that stands out from other macular lesions, particularly dystrophies, which are common causes of misdiagnosis [2]. For instance, in patient #1, biomicroscopy and FFA revealed tiny satellite lesions randomly around the main CNV lesion (Fig. 1). Patient #2 has a similar pattern of tiny pinpoint lesions around the main macular lesion. This peculiar pattern indicates repetitive injury by the laser pointer, which is usually self-inflicted in children and teenagers [1, 3, 12]. Similarly, OCT lesions secondary to laser injury have characteristic features that point to the cause. These include the morphology of outer retinal layer defects/holes (patients #1, 2, 4, and 5), and the anvil-shaped lesion (patient # 2). In addition, the CNV in laser pointer burn is of type II and frequently exhibits the characteristic pitchfork sign, denoting the inciting acute inflammation of the laser burn [19]. Our patients had relatively good visual outcomes despite the dramatic clinical presentation of biomicroscopy and other imaging modalities. The reason could be the young age of the patients included in the study, as 68% of our patients were children and teenagers. At a young age, the Bruch’s membrane is healthy and intact. In the advent of a localized injury produced by a laser pointer, it can provide homeostasis to the RPE and the neurosensory retina, and structural support for RPE cell adhesion and migration to produce healing [18]. In our study, the secondary CNV was resolved using two injections of aflibercept in patient #1, and a single injection in patient #6. The reason for this swift, favorable response could be the localized damage to Bruch’s membrane and the self-limiting nature of the inciting injury. These factors reduce the VEGF load released in response to Bruch’s membrane damage and limit further release after healing, in contrast to the perpetual release of VEGF in response to continuous damage of Bruch’s membrane caused by involutional changes, such as age-related macular degeneration [20]. Therefore, we adopted a customized 1 + pro re nata (PRN) approach, in which the number of injections is determined according to the CNV response without a prior loading dose [20]. The intravitreal injection of aflibercept to treat CNV in our study is an off-label use, particularly in the pediatric population. An important strength in our study is that we employed a multimodal imaging approach, including SS-OCT, SS-OCTA, and FFA, to document the spectrum of pathological features in LPM. SS-OCT and SS-OCTA compensate for the limitations of conventional FFA. These limitations include the inability to achieve depth resolution due to early, profuse leakage of fluorescein dye and light scattering by different retinal layers. The optical properties of SS-OCT and the integrated SS-OCTA enable superior axial resolution and segmentation of different layers of the retina, including the retinal vascular plexuses. These properties include ultra-high-speed image acquisition, a sensitivity roll-off feature, and long wavelength (1050 nm). In addition, the invasive nature of FFA limits its versatility in long-term follow-up and in imaging pediatric patients [21, 22]. In our study, SS-OCT accurately delineated the retinal microstructural pathological changes secondary to laser pointer injury and provided unique morphological features of these lesions. SS-OCTA proved decisive as a non-invasive imaging modality in diagnosing secondary CNV and following up the response to treatment. In patient #5, structural OCT was inconclusive for CNV formation. Patient #5 developed secondary CNV that ended up in a vascularized scar. In this patient, the diagnosis of CNV was only possible by demonstrating the hyperintense signal associated with residual blood flow in a vascularized scarred CNV. Patient #6 had secondary CNV at presentation, SS-OCTA corroborated the structural OCT morphology of secondary CNV by demonstrating high blood flow in the neovascular network. The regression of CNV in patient #6 could only be shown by SS-OCTA, which demonstrated minimal residual blood flow. The limitations of our study include the small number of patients and the wide variation in morphological features, the lack of information available on the causative laser device, its output power, laser wavelength, and important data on the circumstances of injury such as the duration and episodes of exposure and the distance between the device and the eye at the time of occurrence. These limitations hindered performing a statistical correlation between the different features of LPM, the circumstances of the injury, and important parameters such as the visual outcome. From our perspective, LPM is not uncommon but is largely under-reported by patients and misdiagnosed by ophthalmologists. It could produce significant visual handicaps and is a completely preventable condition. The peculiar features of maculopathy demonstrated in the present study should alert the ophthalmologist to the possibility of a laser pointer injury, particularly when detected bilaterally and in a child or a teenager. These findings should prompt rigorous questioning of the child, the parents, or the caretakers to confirm the diagnosis and provide counseling. Public awareness of the condition and liaison between ophthalmologists and legislative and regulatory authorities could help limit the dissemination of these devices.

## Conclusion

SS-OCT effectively depicts a characteristic morphological profile of LPM that helps in early diagnosis and distinguishing LPM from other macular pathologies. SS-OCTA is a non-invasive and reproducible complementary tool in detecting CNV secondary to LPM and monitoring its evolution and response to therapy, particularly in cases where SS-OCT is inconclusive.

## Data Availability

The data that support the findings of this study are available on request from the corresponding author (MM). Access to this data will be granted exclusively to researchers of entities who meet the criteria for access to confidential data.
